# Social cognition in mild cognitive impairment and dementia: A systematic review and meta‐analysis

**DOI:** 10.1002/alz.70076

**Published:** 2025-03-27

**Authors:** Puyu Shi, Hannah Chapman, Lisa Liu, Fern Rodgers, Jasmine Shaw, Gill Livingston, Katherine P. Rankin, Jason D. Warren, Andrew Sommerlad

**Affiliations:** ^1^ Division of Psychiatry University College London London UK; ^2^ Centre for Psychiatry and Mental Health Wolfson Institute of Population Health Queen Mary University of London London UK; ^3^ Camden and Islington NHS Foundation Trust, St Pancras Hospital London UK; ^4^ Department of Neurology Memory and Aging Center University of California San Francisco USA; ^5^ Dementia Research Centre University College London London UK

**Keywords:** Alzheimer's disease, dementia, frontotemporal dementia, mild cognitive impairment, social cognition

## Abstract

Social cognition is impaired in people with dementia but the differences in social cognitive impairment between mild cognitive impairment (MCI) and dementia and its subtypes remain unclear. We therefore aimed to systematically review and meta‐analyze differences in emotion recognition, theory of mind (ToM), and empathy between individuals with MCI and dementia. Across 28 cross‐sectional studies (*n* = 2409), people with MCI had better emotion recognition (Cohen's *d* = 0.69) and ToM (*d* = 0.70) than individuals with Alzheimer's disease (AD) dementia, and larger effect sizes were observed for people with frontotemporal dementia (FTD) (emotion recognition (*d* = 2.09), ToM (*d* = 1.49), but emotional empathy was higher in AD than in MCI in included studies. Our findings suggest a progressive decline of aspects of social cognition across the MCI–dementia continuum. Longitudinal studies should investigate the diagnostic role of social cognition deficits in MCI progression to dementia, and interventions for social cognition in MCI should be developed and tested.

**Highlights:**

- First systematic review and meta‐analysis comparing social cognition between mild cognitive impairment (MCI) and dementia subtypes.
- Findings from 28 studies with 2409 participants show people with MCI outperform those with Alzheimer's disease (AD) and frontotemporal dementia (FTD) in emotion recognition and theory of mind.
- Empathy appears intact in AD dementia, suggesting that this cognitive domain is preserved throughout disease progression.
- Evaluation of social cognition should be built into dementia assessment as it may hold diagnostic value.

## 1 INTRODUCTION

Mild cognitive impairment (MCI) is characterized by cognitive decline that does not affect daily functioning but is greater than expected considering age and education level.^1^ MCI can be classified into amnestic MCI (aMCI), where memory is affected, potentially alongside other cognitive domains such as language, executive functioning, and attention, or non‐amnestic MCI (naMCI), where memory is not affected.^2^ MCI can also be categorized as single‐domain MCI, affecting only one cognitive domain, or multi‐domain MCI, involving impairments in multiple cognitive domains.^2^ MCI is a risk state for dementia with an annual progression rate of 10%–15% from aMCI to Alzheimer's disease (AD) dementia^3^, ^4^, ^5^, ^6^ and of 5%–10% from all‐cause MCI to all‐cause dementia^6^, ^7^, ^8^; therefore represents a potential window for early diagnosis and intervention, meaning it is crucial to distinguish which cases will progress to dementia from those that will remain stable or even improve. Although most research has typically focused on traditional cognitive domains such as memory, language, executive function, and attention, less is known about social cognition impairment in MCI. In addition, social cognition is recognized as a core cognitive domain equal in importance to other cognitive domains in Diagnostic and Statistical Manual of Mental Disorders, 5th Edition (DSM‐5) diagnostic criteria for neurocognitive disorders including dementia.^9^ Moreover, the potential predictive value of social cognition impairment in MCI for progression to dementia remains unclear.

Socially inappropriate behaviors are problems that can be seen in some individuals with dementia, and cause distress to patients and their families.^10^ These problems may partly be due to general cognitive decline as well as specific social cognition impairments.^11^ Social cognition is the ability to perceive and process social information from others, which involves complex cognitive processes starting with the perception of social cues that distinguish others as ``living persons’’ from objects. These cues are then integrated into higher‐level processes to resonate with others' emotional states and to understand their intentions and behaviors (see^12^ for a comprehensive review). Social cognition is crucial for successful social interactions and maintaining social relationships,^13^ and the key domains include emotion recognition, theory of mind (ToM)/mentalizing, and empathy. Although emotion recognition is a fundamental aspect of social perception, both ToM and empathy are more complex processes for facilitating social understanding.^12^

Emotion recognition is a fundamental aspect of social perception, enabling people to identify others’ emotions through facial expressions, gestures, and voices.^14^ Among these, human faces hold the unique salience for emotion recognition and are processed differently from objects, relying on rapid holistic coding (in contrast to the part‐based coding used for objects) of the relationships between facial features (e.g., eyes, nose, and mouth) into an integrated representation.^15^ The facial expressions of six basic emotions—happiness, anger, sadness, fear, disgust, and surprise—are considered universal and can be recognized by all humans.^16^ Although facial expressions are a primary source of emotional information, other cues such as body movements and vocal tone also play a role in providing a more efficient and holistic recognition of emotions.^17^, ^18^ In addition, external and contextual factors, such as an individual's emotional experiences and current emotional state, influence emotion recognition.^19^ Although basic emotions are universally recognized, higher accuracy is observed when emotions are both expressed and recognized by individuals who share the same cultural background.^20^

ToM or mentalizing, refers to the ability to understand others’ thoughts, beliefs, and intentions even when they differ from one's own.^21^ ToM can be divided into two components based on the type of mentalizing inference: affective and cognitive.^22^, ^23^ Affective ToM involves understanding and inferring others’ emotions and feelings, focusing on the affective states of others, whereas cognitive ToM refers to the ability to reason about others’ beliefs, intentions, and knowledge, often requiring perspective‐taking. Cognitive ToM is also considered a prerequisite for affective ToM, as understanding others’ emotions often depends on first interpreting their mental states.^24^

Empathy is the capacity to understand and share the feelings of others. It comprises two components: emotional empathy, which involves feeling others' emotions by affective sharing through emotional mirroring, and cognitive empathy, which involves understanding another's emotional experiences through cognitive evaluation of their internal state.^25^, ^26^ The emotional and cognitive components of empathy interact dynamically to enable effective empathic responses, whereas contextual cues and previous experiences will also affect one's ability to infer and respond to others' emotions.^27^

Early social cognitive decline is a key diagnostic feature of behavioral variant frontotemporal dementia (bvFTD),^28^, ^29^ and social cognitive impairments can be the earliest clinical symptoms and may precede formal diagnosis by years in familial bvFTD.^30^, ^31^ For dementia caused by AD, previous meta‐analyses have found that emotion recognition, ToM, and cognitive empathy are impaired compared to healthy older adults, whereas affective empathy appears to remain intact.^32^, ^33^, ^34^ Reviews have also suggested impaired emotion recognition in samples of people with predominantly aMCI^35^ and all‐cause MCI^36^ compared to healthy older adults and impaired ToM task performance in MCI compared to healthy older adults.^37^

The previous literature has shown that social cognitive performance differs between people with dementia and healthy older adults, as well as between people with MCI and healthy older adults. However, social cognition across the important clinical distinction between those with MCI and progressive dementia has not been investigated extensively. Some reviews have compared emotion recognition^32^ and ToM between MCI and AD dementia,^18^ but these reviews have considered only individual social cognitive domains or focused on the AD dementia subtype only.^32^, ^33^, ^34^ No study has investigated impairment across different social cognitive domains and clarified the social cognitive profile when comparing MCI to various dementia subtypes. Understanding the differences in social cognition profiles between MCI and dementia has clinical significance, as it may help clarify the predictive role of social cognition in the progression from MCI to dementia. Our review therefore aims to compare the social cognitive differences, measured by emotion recognition, ToM, and empathy, between people with MCI and those with dementia.

## 2 METHODS

Our systematic review and meta‐analysis were pre‐registered with International Prospective Register of Systematic Reviews (PROSPERO). (https://www.crd.york.ac.uk/prospero/display_record.php?ID=CRD42023489359).

### 2.1 Search strategy

We conducted a literature search across the following electronic databases: MEDLINE, PsycINFO, EMBASE, and CINAHL. There were no restrictions on the language of publication, country of origin, or publication date of the studies. The date of last search was January 22, 2024. Our search terms (presented in full in Appendix A) were determined based on the key concepts relevant to our research question on comparing social cognition in people with MCI and people with dementia:

1. Concept 1: ``Dementia'' (all subtypes)
2. AND Concept 2: ``mild cognitive impairment (MCI)’’
3. AND Concept 3: ``social cognition'', OR ``emotion recognition'', OR `` theory of mind'', OR ``empathy''.

For these concepts, we searched for synonyms and related topics and mapped the searches to subject headings. In addition, we hand‐searched the reference lists of included studies and relevant systematic reviews to identify additional relevant articles.

### 2.2 Eligibility

Two types of studies were eligible for inclusion: those which cross‐sectionally compared social cognition (emotion recognition, empathy, and ToM) between people with MCI and people with dementia, or cohort studies that examined the association of social cognition performance with the progression from MCI to dementia. All subtypes of MCI and dementia were considered. For cross‐sectional studies, participants were required to have a clinical diagnosis of MCI or dementia based on recognized, validated diagnostic criteria. For cohort studies, participants needed to have a clinical diagnosis of MCI at the beginning of the study, and dementia at follow‐up, as assessed using relevant standardized diagnostic criteria. Examples of diagnostic criteria for dementia include the National Institute on Aging–Alzheimer's Association (NIA‐AA) criteria for AD and the DSM‐5 for major neurocognitive disorder.^38^, ^39^ Examples of diagnostic criteria for MCI include Petersen's criteria, NIA‐AA, and International Working Group (IWG)/Winblad Criteria.^3^, ^40^, ^41^ People with subjective cognitive decline without a clinical diagnosis of MCI or those described as MCI solely based on Mini‐Mental Status Examination (MMSE) scores or cognitive ratings were not included.

Eligible studies must have assessed social cognition using self‐rated or observer/informant‐rated scales or experimental tasks.

### 2.3 Data extraction and synthesis

The selection and analysis of data from the eligible studies followed the Preferred Reporting Items for Systematic Reviews and Meta‐Analyses (PRISMA) guidelines. The search results were de‐duplicated and imported into Covidence. Titles and abstracts were first screened for relevance. For studies included in full‐text screening, two reviewers independently reviewed each study to assess eligibility, and any discrepancies were discussed amongst the team and resolved through consultation with a third reviewer. Reasons for excluding studies were recorded and presented in a PRISMA diagram (Appendix B). The average Cohen's kappa for inter‐rater reliability among the six involved reviewers was 0.80, indicating substantial agreement.

For each included study, two independent researchers extracted data into a data extraction form. We collected study characteristics including publication date, first author, study design, sample size, age, dementia classification criteria and subtypes, MCI classification criteria and subtypes, severity of cognitive impairment, social cognition assessment tools used, statistical methods, and results. If data were missing, inaccessible, or unclear, the study authors were contacted for additional information.

### 2.4 Quality assessment

We used a modified version of the Newcastle‐Ottawa Scale (NOS) to assess the quality of the included studies.^42^ Domains considered in the quality assessments for both types of studies were: representativeness of the sample, response rate, sample size, assessment of dementia/MCI, assessment for social cognition, and comparability of people with MCI and people with dementia based on the design or analysis (whether age and cognition were adjusted in the analysis). The total points earned by each study were calculated for its overall risk of bias. The total score ranges from 0 to 9, with a lower score indicating a higher risk of bias.

### 2.5 Analysis

The final selected studies were summarized narratively to report the differences in the three social cognition domains (emotion recognition, ToM, and empathy) for people with MCI and people with dementia. Given the heterogeneity in defining these domains and their synonyms in the current literature, we employed the following approach to classify social cognition assessments.

For emotion recognition, we included tasks that assessed lower‐level social perception by identifying emotions from simple social cues (e.g., facial expressions, facial features, or voice) without contextual information.^43^ Although the Reading the Mind in the Eyes Test (RMET) is often used as a ToM measure,^44^ we classified it under emotion recognition for this review. This decision was based on its similarity to classic emotion recognition paradigms (e.g., Ekman) and its focus on identifying emotions from the eye region, the most salient area for facial emotion recognition without consideration of underlying mental state.^45^, ^46^ Furthermore, evidence suggests RMET performance is more influenced by alexithymia (difficulty identifying and describing emotions) than by ToM deficits.^47^

We considered ToM and empathy as higher‐order cognitive processes requiring social understanding and contextual interpretation. However, substantial heterogeneity and ambiguity exist in their definitions and subdomains. For example, affective ToM and cognitive empathy are sometimes used interchangeably in the literature, although some definitions differentiate them, suggesting that cognitive empathy involves emotional awareness.^25^ Given this heterogeneity, we classified ToM and empathy based on the original articles’ definitions. We conducted separate analyses for cognitive empathy and emotional empathy because all included studies assessing empathy used the Interpersonal Reactivity Index (IRI),^48^ which provides distinct subscales for these components. However, such subgroup analyses were not feasible for ToM due to the diversity of assessments and the lack of consistent separation between affective and cognitive ToM in the included studies.

We performed meta‐analyses of the standardized mean differences in social cognition between MCI and dementia, where two or more studies assessed the same domain of social cognition using similar paradigms. The mean scores and standard deviations (SDs) were used in the meta‐analysis. If such information was not available, we calculated or transformed the existing data to obtain these where applicable (Appendix C). A random‐effects model was used to pool the effect sizes, and the overall pooled effect size and its 95% confidence interval (CI) were calculated to determine the magnitude of the effect. Heterogeneity among studies was assessed using the I^2^ index. An I^2^ <25% is considered low heterogeneity, between 25% and 50% is considered moderate, and >50% as high heterogeneity.^49^ All statistical analyses were conducted using Stata *MP* version 18.^50^

Separate meta‐analyses were conducted by grouping studies according to the social cognition domains of interest: emotion recognition, ToM, and empathy. For each domain, subgroup analyses were performed for all‐cause MCI versus AD dementia and all‐cause MCI versus frontotemporal dementia (FTD) comparisons when applicable. Comparisons with other dementia subtypes were not feasible due to the small number of cases in the included studies (see Section 3.1). It was also not feasible to conduct subgroup analyses to compare dementia stages and the social cognition impairment profiles in each domain due to a limited number of studies that compared social cognition performance in different dementia stages (see Section 3.1). For these studies, we calculated the average social cognition performance scores across all AD stages and included them in the MCI versus AD dementia analyses to represent the overall AD dementia social cognition profile.

In addition, we conducted subgroup analyses to compare aMCI versus AD dementia, as aMCI is often considered a prodromal stage of AD dementia. Comparisons between aMCI and non‐aMCI with other dementia subtypes were not possible due to the small number of studies reporting non‐aMCI diagnoses and the small number of other dementia subtypes reported in the included studies.

We excluded studies from meta‐analyses if dementia subtypes were not specified, if mean scores were unavailable, or if participants potentially overlapped with those in another included study (in such cases, the study with the larger sample size was included). A full list of excluded studies and their reason for exclusion is provided in Appendix C.

## 3 RESULTS

### 3.1 Study selection and characteristics

The PRISMA diagram (Appendix B) summarizes the study selection and reasons for exclusion. Of the 2490 identified studies, 28 were included in this review.^51^, ^52^, ^53^, ^54^, ^55^, ^56^, ^57^, ^58^, ^59^, ^60^, ^61^, ^62^, ^63^, ^64^, ^65^, ^66^, ^67^, ^68^, ^69^, ^70^, ^71^, ^72^, ^73^, ^74^, ^75^, ^76^, ^77^, ^78^

All the included studies conducted cross‐sectional comparisons of social cognition differences between dementia and MCI groups; we did not identify any cohort studies examining progression from MCI to dementia. Among the 28 studies, emotion recognition was the most frequently assessed social cognitive domain (23 studies), followed by ToM (9 studies) and empathy (5 studies) (Table 1).^79^, ^80^, ^81^, ^82^, ^83^, ^84^, ^85^, ^86^, ^87^, ^88^, ^89^ One study had a low‐quality rating of 2, and all others had a moderate quality rating ranging from 4 to 6.

**TABLE 1:** Study characteristics and quality rating.

First author, year of publication	Quality rating	Country	Sample size and diagnosis	Diagnostic criteria used	Setting of recruitment	Mean age (SD)	No. and % female	MMSE, mean (SD)	Social cognitive domain(s) studied
Hayashi 2021	6	Japan	Dementia: 63 (AD: 44, non‐specified: 19) MCI: 92	DSM‐5 NIA‐AA	Memory Clinic of Okayama University Hospital	77.5 (7.3) 75.7 (8.5)	35, 56% 56, 61%	19.9 (3.2) 26.2 (2.4)	Emotion recognition
Giacomucci 2024	5	Italy	AD: 80 MCI: 83	NIA‐AA NIA‐AA	Not described	71.0 (7.0) 71.4 (8.3)	44, 55% 50, 50.2%	19.3 (5.8) 28.9 (1.3)	Empathy emotion recognition
Chander 2025	4	Australia	Dementia: 53 MCI: 129	DSM‐IV IWG criteria	Community	88.6 (4.25) 87.3 (4.07)	27, 54% 85, 65.9%	25.88(3.87) 28.86(1.45)	Empathy emotion recognition
Eramudugolla 2022	4	Australia	Dementia: 23 (AD: 20; VD: 2; PD: 1) MCI: 132	DSM‐IV IWG criteria	Community	75.9 (1.3) 75.2 (1.6)	9, 39.1% 61, 46.2%	Not reported	Emotion recognition
Garcia‐Casal 2019	4	Spain	AD: 84 aMCI: 59	DSM‐4‐TR, NINCDS‐ADRDA IWG criteria	Outpatient memory clinics at Burgos University Hospital	78.3 (5.8) 77.6 (5.0)	32, 38% 27, 47%	21.6 (3.6) 24.0 (2.4)	Emotion recognition
Sturm 2013	4	USA	AD: 64 MCI: 62	NINCDS‐ADRD Modified criteria	no description	64.4 (11.3) 69.4 (10.6)	30, 46.9% 33, 53.2%	20.7 (5.2) 28.6 (1.5)	Emotional contagion (empathy)
Weiss 2008	5	Austria	Mild AD: 30 Moderate AD: 23 Single‐domain aMCI: 21 Multi‐domain aMCI: 30	DSM‐3, NINCDS‐ARDRA Petersen criteria, IWG criteria	Outpatient clinics at the Department of Psychiatry in Innsbruck	76.7 (8.0) 80.1 (6.2) 72.8 (6.5) 74.3 (7.0)	22, 73.3% 16, 69.6% 15, 71.4% 20, 66.7%	22.5 (1.5) 16.3 (2.7) 27.0 (1.0) 26.0 (1.1)	Emotion recognition
Spoletini 2008	4	Italy	Mild AD: 50 aMCI: 50	NINCDS‐ARDRA Petersen criteria	Outpatient clinics	72.7 (6.9) 71.2 (7.5)	25, 50% 23, 46%	22.0 (3.3) 26.7 (2.5)	Emotion recognition
Strijkert 2022	5	Netherlands	AD: 45 aMCI: 47	NIA‐AA Petersen criteria	Memory clinic	71.6 (1.2) 67.5 (1.2)	22, 48.89% 20, 42.55%	24.9 (0.4) 27.2 (0.4)	Emotion recognition
Ferrer‐Cairols 2023	4	Spain	Mild AD: 20 Mild dementia‐non AD: 13 (FTD: 6; non‐specified: 7) MCI‐AD: 25 MCI‐non AD: 34	NIA‐AA NIA‐AA	Neurology unit of the University and Polytechnic Hospital La Fe	73 (70–75) 67 (64‐75) 73 (66–75) 64 (60‐68) ^a^	14, 70% 6, 46.2% 14, 56% 20, 58.8%	Not reported	Emotion recognition
Giacomucci 2022	6	Italy	AD: 46 MCI: 41	NIA‐AA NIA‐AA	No description	69.2 (6.2) 71.8 (8.1)	24, 52.2% 27, 68.9%	18.8 (5.5) 26.6 (2.6)	Emotion recognition, empathy
Amlerova 2022	5	Czech Republic	AD: 36 aMCI: 43	EFNS‐ ENS guidelines Petersen criteria	Memory clinic of Motol University Hospital	74.5 (6.5) 74.5 (6.1)	36, 69.4% 43, 60.5%	22.8 (2.6) 26.6 (2.6)	Emotion recognition
Park 2017	6	South Korea	AD: 32 FTD: 13 (bvFTD: 8; semantic dementia: 5) MCI: 32	DSM‐IV, NINCDS‐ADRDA Neary criteria DSM‐IV, Petersen criteria	SMG‐SNU Boramae Medical Center and Donjak‐Gu Center for Dementia	76.8 (8.5) 71.9 (3.6) 74.3 (4.6)	17, 53.12% 9, 69.23% 21, 65.63%	19.0 (3.4) 18.6 (7.2) 24.7 (2.4)	Emotion recognition
Yamaguchi 2019	4	Japan	Mild AD: 34 Moderate AD: 17 MCI: 25	NINCDS‐ARDRA Petersen criteria	Outpatient clinic	79.5 (6.1) 82.4 (5.1) 76.4 (6.6)	19, 55.9% 15, 88.2% 17, 68%	19.6 (3.5) 13.6 (3.5) 25.4 (2.0)	Theory of Mind
Sheardova 2014	5	Czech Republic	Mild AD: 29 Single‐domain aMCI: 13 Multi‐domain aMCI: 30	NINCDS‐ADRDA Petersen criteria	Memory Clinic of the Motol University Hospital	74.4 (8.4) 72.6 (7.7) 71.9 (9.2)	17, 58.6% 9, 69.2% 13, 43.3%	19.8 (3.3) 27.0 (2.3) 26.0 (2.9)	Emotion recognition
Maki 2013	6	Japan	Mild AD: 30 aMCI: 42	NINCDS‐ADRDA IWG criteria	Outpatient clinics	78.0 (7.2) 74.0 (5.4)	24, 80% 24, 58.1%	21.4 (4.0) 25.8 (1.7)	Theory of Mind
Henry 2009	4	Australia	Dementia: 34 MCI: 38	DSM‐IV Petersen criteria	Community and a Memory clinic	79.4 (6.1) 78.7 (4.5)	18, 53% 19, 50%	26.0 (3.6) 27.9 (1.5)	Emotion recognition
Strijkert 2023	5	Netherlands	AD: 31 aMCI: 37	NIA‐AA Petersen criteria	Memory Clinic of the Giatrics Department of the University Medical Center Groningen	71.1 (8.4) 69.6 (7.7)	16, 51.6% 20, 54.1%	25, 20–30 27, 22‐30 (median and range)	Emotion recognition
Yamaguchi 2012	4	Japan	Mild AD: 36 Moderate AD: 14 aMCI: 12	NINCDS‐ADRDA Petersen criteria	Outpatient clinics	72.9 (5.8) 81.0 (5.5) 74.4 (5.0)	24, 66.7% 13, 92.9% 6, 50%	19.9 (3.3) 14.1 (3.1) 25.0 (3.2)	Theory of Mind
Henry 2012	4	Australia	Dementia: 26 MCI: 36	DSM‐IV IWG criteria & Peterson criteria	Community and a Memory clinic	80.0 (6.3) 78.3 (4.1)	10, 38.5% 17, 47.2%	25.9 (3.1) 27.6 (1.9)	Emotion recognition
Kessels 2021	4	Netherlands	AD: 29 aMCI: 31	NIA‐AA NIA‐AA	Department of Medical Psychology of Ziekenhuis Groep Twente (a General Hospital) and a Mental Health Care Facility	76.8 (6.5) 75.4 (6.6)	18, 62.07% 13, 41.94%	19.2 (4.7) 24.2 (2.8)	Emotion recognition, Theory of Mind
Schild 2021	4	Germany	AD: 30 aMCI: 28	NIA‐AA NIA‐AA	Center for Memory Disorders at the University of Cologne	72.1 (8.7) 73.6 (5.8)	14, 46.67% 14, 50%	25.3 (2.7) 26.6 (2.0)	Emotion recognition, Theory of mind
Cardenas 2021	6	Spain	Moderate AD: 32 MCI: 24	DSM‐IV; NINCDS‐ADRDA Petersen criteria	Community	82. 8 (5.4) 83.0 (5.6)	No information for subgroups	18.7 (3.2) 27.2 (1.6)	Emotion recognition
Yildirim 2020	5	Türkiye	Mild AD: 18 MCI: 31	NIA‐AA Petersen criteria	Istanbul University, Istanbul Faculty of Medicine, Department of Neurology, Behavioral Neurology, and Movement Disorders Unit	69.7 (8.8) 64.5 (8.7)	7, 38.89% 11, 35.48%	Not reported	Emotion recognition, Theory of Mind
Formica 2020	2	Italy	AD: 24 FTD:14 MCI: 10	no information	no description	78.4 (6.6) 74.3 (4.7) 66.6 (6.7)	No information	17.0 (3.6) 17.5 (4.6) 23.5 (1.0)	Theory of Mind, Emotion recognition
Dodich 2016	5	Italy	AD: 12 bvFTD: 20 aMCI: 15	NIA‐AA IFTDC criteria Petersen criteria	Department of Clinical Neurosciences, Vita‐Salute San Raffaele University and San Raffaele Scientific Institue	73.2 (10.1) 66.8 (8.7) 73.1 (6.2)	5, 42% 8, 40% 5, 33.3%	21.5 (3.9) 24.8 (3.4) 25.6 (2.3)	Theory of Mind, empathy
Bediou 2009	4	France	Mild AD: 10 FTD: 10 aMCI: 10	NINCDS‐ADRDA Neary criteria Petersen criteria	Neurological Hospital of Lyon	72.0(9.0) 67.0(7.0) 73.0(9.0)	5, 50% 5, 50% 5, 50%	21.1 (1.6) 24.1 (3.8) 27.0 (1.7)	Emotion recognition
Yao 2022	4	Japan	Mild AD: 11 MCI: 11	DSM‐5 DSM‐5	Outpatient Memory Clinic of Kobe University Hospital	81.1 (5.3) 78.3 (5.2)	7, 63.6% 8, 72.7%	22.2 (2.4) 25.0 (1.9)	Theory of Mind

*Note*: Diagnostic criteria used across the studies:.

AD: National Institue on Aging ‐ Alzheimer's Association (NIA‐AA),^38^ National Institute of Neurological and Communicative Disorders and Stroke ‐ Alzheimer's Disease and Related Disorders Association (NINCDS‐ADRDA),^79^, ^80^ Diagnostic and Statistical Manual of Mental Disorders (DSM),^39^, ^81^, ^82^ European Federation of Neurological Societies ‐ European Neurological Society (EFNS‐ENS) guidelines.^83^

FTD: Neary criteria for FTD,^28^ International Behavioural Variant FTD Criteria Consortium (IFTDC) criteria.^84^

MCI: NIA‐AA,^2^ Petersen criteria,^1^, ^3^, ^41^, ^85^, ^86^, ^87^, ^88^ International Working Group (IWG) criteria,^40^ DSM,^39^ Modified criteria.^89^

Abbreviations: AD, Alzheimer's disease dementia; FTD, frontotemporal dementia; MCI, mild cognitive impairment; MMSE, Mini‐Mental State Examination.

^a^ Median and age range were reported.

The study characteristics and quality rating are summarized in Table 1. There were 2409 participants, including 1136 people with dementia and 1273 with MCI. Among participants with dementia, 931 were diagnosed with AD dementia, with 279 of these diagnoses supported by cerebrospinal fluid (CSF) biomarkers indicating positive amyloid beta (Aβ). For the stages of AD dementia, seven studies focused on mild AD dementia only,^53^, ^54^, ^55^, ^56^, ^60^, ^66^, ^71^ one study focused on moderate AD dementia,^67^ three studies reported social cognition performance in both mild and moderate AD dementia,^62^, ^70^, ^72^ and 13 studies did not specify the AD dementia stages of their participants.^51^, ^52^, ^57^, ^58^, ^59^, ^61^, ^63^, ^64^, ^65^, ^69^, ^73^, ^74^, ^76^ Sixty‐three participants had FTD, including 28 with bvFTD, five with semantic FTD, and 30 with unspecified FTD subtype. In addition, two participants had vascular dementia (VaD), one had Parkinson's disease (PD) dementia, and 139 had unspecified dementia. The stages of FTD and other dementia subtypes were not reported in these studies.

Of the people with MCI, 629 were diagnosed with aMCI, 67 with non‐aMCI, and 518 did not have the MCI subtype described. One study classified patients with MCI based on their relationship to AD biomarkers, identifying 25 as AD‐MCI and 34 as non‐AD‐MCI. Of participants with aMCI, where subtypes were specified, 114 participants had single‐domain aMCI and 109 had multi‐domain aMCI. AD dementia was diagnosed using the National Institute of Neurological and Communicative Disorders and Stroke ‐ Alzheimer's Disease and Related Disorders Association (NINCDS‐ADRDA) criteria (*n* = 10),^79^, ^80^ NIA‐AA criteria (*n* = 9),^22^ and DSM (*n* = 10 studies, with variations in versions used).^39^, ^81^, ^82^ Specific diagnostic criteria were applied for FTD.^28^, ^84^ For MCI, the most frequently adopted criteria were the Petersen criteria (*n* = 15 studies, with variations in versions referenced),^1^, ^3^, ^41^, ^85^, ^86^, ^87^, ^88^ NIA‐AA (*n* = 6 studies),^2^ and the IWG/Winblad criteria (*n* = 6 studies).^40^

The mean age of participants from the included studies ranged from 64 to 83 years. Sex distribution was reported in 26 studies, with female participants comprising 54.2% of the dementia group and 52.6% of the MCI group.

In 25 studies that provided information on the setting of recruitment, most studies (*n* = 16) recruited participants from medical settings (e.g., hospitals and memory clinics).^52^, ^54^, ^55^, ^56^, ^57^, ^58^, ^59^, ^60^, ^61^, ^62^, ^63^, ^66^, ^69^, ^70^, ^71^, ^72^ Three studies mentioned recruiting participants from university departments or research centres^53^, ^73^, ^74^; three studies recruited participants from community settings; and two studies were from a mixture of community settings and memory clinic settings.^68^, ^78^ Among the 24 studies that reported MMSE scores, the mean MMSE score for participants with AD dementia ranged from 19.6 to 25.9 for mild AD dementia and 13.6 to 18.7 for moderate AD dementia. The mean MMSE score for FTD participants ranged from 18.6 to 24.8. For MCI participants, mean MMSE scores ranged from 23.5 to 28.9.

### 3.2 Assessments of social cognition

#### 3.2.1 Emotion recognition

The assessment methods adopted by the included studies can be classified broadly into facial and non‐facial emotion recognition paradigms (Table 2).^90^, ^91^, ^92^, ^93^, ^94^, ^95^, ^96^, ^97^, ^98^, ^99^, ^100^, ^101^, ^102^, ^103^, ^104^, ^105^ Most studies (*n* = 21) assessed emotion recognition using pictures of facial expression stimuli (e.g., Ekman 60 Faces Test) or parts of facial expressions (e.g., RMET, which shows only the eye region). These tasks typically involved presenting participants with faces displaying emotions, such as anger, sadness, happiness, fear, disgust, and surprise, and asking them to identify the facial emotion displayed from a list of labels. Most tasks relied on faces displaying expressions with a negative valence. Some paradigms manipulated the valence/intensity of the facial emotion expression. For example, the Penn Emotion Recognition Task adopted by three studies included facial stimuli that varied in ``mild’ to ``extreme’ facial emotional expressions.^55^, ^62^ Two other studies used a similar approach with different tasks.^56^, ^67^, ^68^

**TABLE 2:** Assessments of social cognition adopted by the included studies.

Study	Task	Task type	Description
**Emotion recognition**			
Giacomucci 2024	EK‐60F	**Ekman** ^46^	**Stimuli**: 25–60 black and white pictures from the Ekman and Friesen series of Pictures of Facial Affect, showing faces displaying one of the six basic emotions: anger, sadness, happiness, fear, disgust, and surprise. **Procedure**: Participants were asked to indicate which of the six basic emotions best represented the facial expression shown.
Giacomucci 2022			
Strijkert 2022			
Strijkert 2023			
Henry 2012	EK‐36F		
Henry 2009			
Park 2017	EK‐35F		
Sheardova 2014	EK‐25F		
Spoletini 2008	PERT	**PERT** ^90^, ^91^	**Stimuli**: Color photographs of facial expressions showing emotions (happy, sad, angry, fearful, disgusted, neutral). Each emotion had low‐intensity and high‐intensity expressions, plus neutral expressions. **Procedure**: Participants selected the labels that match for the displayed facial expression, and rated the emotional valence of each expression.
Weiss 2008			
Chander 2025	RMET	**RMET** ^44^	**Procedure**: Participants are presented with each photograph and asked to indicate the emotional description (e.g., “angry,” “amused”) that best matches the expression in the eyes. **Stimuli**: 36 (RMET) or 32 (RMET‐32) black and white photographs of faces only showing the eye regions.
Eramudugolla 2022			
Ferrer‐Cairols 2023			
Formica 2020			
Schild 2021			
Yildirim 2020	RMET‐32		
Chander 2025	The emotion recognition task	**Morphed facial expression tasks** ^92^, ^93^, ^94^	**Stimuli**: Facial images of basic emotions such as anger, sadness, surprise, happiness, fear, and disgust. Each emotion was morphed with a neutral face in 20% steps. **Procedure**: Participants labeled the displayed emotion using an alternative forced‐choice response.
Kessels 2021			
Bediou 2009	Facial expression task		
Hayashi 2021	Facial expression recognition test		
Garcia‐Casal 2019^a^	Affect‐GRADIOR emotion recognition test	**Other facial emotion recognition tasks** ^57^, ^95^, ^96^	**Stimuli**: Color photographs of professional actors expressing six basic emotions (happiness, sadness, anger, fear, disgust, surprise) and a neutral expression. **Procedure**: Participants view each photograph and select the emotion label that best describes the facial expression shown.
Cardenas 2021	Florida affect battery‐task 3 and 4; emotion Labeling Task		**Stimuli**: Photographs of facial expressions. **Procedures**: Facial Affect Naming Task (FAB‐3): Participants chose from emotional word categories (and intensity) best corresponding to the expression in the photograph. Facial affect selection task (FAB‐4): From five photographs of each screen, the participants selected which of five photographs displayed the indicated emotion. Emotion labeling: Participants labeled emotional facial expressions.
Schild 2021	Karolinska directed emotional faces		**Stimuli**: Color photographs of faces displaying happiness, sadness, fear, disgust, anger, surprise or a neutral expression. Each emotion included stimuli with higher or lower chance of recognition based on normative data. **Procedure**: Participants selected the word that best matched the facial expression.
Amlerova 2022	Emotion prosody task	**Non‐facial emotion recognition tasks** ^97^, ^98^, ^99^	**Stimuli**: Short audio recordings spoken by one male and one female performer that included sentences with neutral semantic meaning (e.g., “The table has four legs”). Each recording was presented with an emotionally charged voice representing one of five emotions: happiness, sadness, fear, disgust, and anger. **Procedure**: Participants listened to the recordings and selected the appropriate emotion from a list of emotions.
Cardenas 2021			
Henry 2012	Point light animation task		**Stimuli**: 12 point‐light animations of a whole‐body figure displaying emotions (anger, fear, sadness, happiness) **Procedure**: Participants select which emotion label best described the emotion portrayed by the point‐light walker.
**ToM**			
Kessels 2021	Four first‐order belief ToM stories	**Text‐based** ^100^, ^101^, ^102^, ^103^	**Stimuli**: Four short stories including elements requiring inference about the characters’ feelings, ideas, or knowledge. **Procedure**: Participants read each story aloud, then answered a factual question and a first‐order ToM question requiring them to infer the characters thoughts.
Maki 2013	MSST		**Stimuli**: 10 sentences (5 metaphoric and 5 sarcastic), selected from Japanese language textbooks for first, second, and third grades in elementary school. **Procedure**: For each sentence, participants chose the correct interpretation from five multiple‐choice options. The incorrect choices included a literal interpretation, an answer associated with part of the sentence, a misunderstanding of the sentence, and not knowing.
Yao 2022	MSCEIT‐ME		**Emotion management tasks** **Stimuli**: Five stories, each with four possible responses. **Procedure**: Participants read a series of stories where a character experiences a specific emotion. They judged which option would be most effective to achieve the desired emotional outcome for the character, e.g., to reduce anger or prolong joy. **Emotional relationships task** **Stimuli**: Three items, each with three possible responses. **Procedure**: Participants read scenarios involving the management of another person's emotions. They judged which of the three response options is most effective for one person to influence or manage another person's feelings.
Yildirim 2020	Faux pas recognition test		**Stimuli**: Ten faux pas stories involving a character unintentionally saying something offensive or inappropriate; 10 stories about socially appropriate interactions. **Procedure**: Participants read each story and were asked whether a faux pas was committed. If so, participants then answered follow‐up questions assessing their understanding of the situation.
Dodich 2016	SET	**Cartoon‐based** ^70^, ^72^, ^104^, ^105^	**Stimuli**: Comic strips **Procedure**: Participants were presented with comic strips and asked to describe each strip and propose a potential ending. After this, they selected an ending from three provided options. These tasks assessed intention attribution, emotion attribution, and causal inference.
Formica 2020			
Schild 2021	Theory of mind picture stories task		**Stimuli**: Six picture stories, each consisting of four colored cartoons, involving interactions where characters cooperate or deceive each other to reach a goal. **Procedure**: Participants rearranged the randomly mixed pictures into a meaningful order. If the order was incorrect, the investigator would correct it. After arranging the pictures, participants answered 2–5 open questions, including a control question to verify story comprehension. The task assessed various levels of ToM complexity (first, second, and third order) and recognition of cheating, reciprocity, and deception.
Yamaguchi 2012^a^	Pitfall task		**Stimuli**: A single‐frame cartoon depicting a scene with a misbehaving child hiding behind a tree imagining another person falling into a pitfall. **Procedure**: Participants answered seven questions about the cartoon to see if they could interpret the intentions of the characters.
Yamaguchi 2019^a^	Comprehension of cartoon picture (false belief test)		**Stimuli**: Single‐frame monochromatic cartoons depicting (1) a man intending to deceive a woman by drawing her attention to the TV to take a cookie from her, (2) the same scene without any intention to deceive. **Procedure**: Participants were asked nine questions for each cartoon to assess their understanding and reasoning about the characters’ intentions, gaze, and pointing
**Empathy**			
Chander 2025	IRI‐EC; IRI‐PT	**IRI** ^48^	**Stimuli**: 28‐item questionnaire with four 7‐item subscales: Two subscales assessed cognitive empathy: fantasy, perspective‐taking Two subscales assessed emotional empathy: empathic concern, personal distress **Procedure & Scoring**: Caregivers rated each statement based on the participant's current behaviors on a scale from 1 (does not describe at all) to 5 (describes very well).
Dodich 2016	IRI		
Giacomucci 2022	IRI		
Giacomucci 2024	Interpersonal reactivity index		
Sturm 2013	IRI‐PD		

Abbreviations: EK‐60F, Ekman 60‐Faces Test; IRI, Interpersonal Reactivity Index; IRI‐EC, Interpersonal Reactivity Index‐Empathic Concern subscale; IRI‐PD, Interpersonal Reactivity Index‐Personal Distress subscale; IRI‐PT, Interpersonal Reactivity Index‐Perspective Taking subscale; MSCEIT‐ME, Mayer‐Salovey‐Caruso Emotional Intelligence Test's Managing Emotions Component; MSST, Metaphoric and Sarcastic Scenario Test; PERT, Penn Emotion Recognition Test; SET, Story‐based Empathy Task.

^a^ Studies that used a self‐developed assessment for social cognition.

Three studies assessed emotion recognition using non‐facial stimuli,^59^, ^67^, ^68^ where participants had to identify the emotion portrayed in a sound recording of an emotionally charged voice or a point‐light animation of a whole‐body figure.

#### 3.2.2 ToM

ToM was assessed in nine studies using diverse methods, adopting both text‐ and cartoon‐based (still image) stimuli. Text‐based assessments involved presenting participants with a social scenario followed by questions evaluating their ability to understand the characters’ intentions or feelings, comprehend socially appropriate behaviors (such as in Faux Pas Recognition Test),^53^, ^100^ and interpret metaphorical and sarcastic sentences beyond their literal meaning.^71^ Cartoon‐based assessments were adopted in five studies. Three of these studies used false belief tasks^70^, ^72^, ^74^ involving scenarios where one character deceives another. These tasks assessed participants' ability to distinguish other's mental states from their own by recognizing that the deceived character holds beliefs that differs from reality and from the participant's own knowledge. Two studies employed the Story‐based Empathy Test (SET),^73^, ^76^ where participants were presented with three cartoon stories, and asked to describe each strip and select an appropriate ending from three provided options, aiming to assess participants’ ability to attribute the intention and emotion of the characters.

#### 3.2.3 Empathy

Studies assessing empathy used an informant‐rated version of the IRI,^48^, ^51^, ^64^, ^73^, ^78^ a 28‐item scale divided into four 7‐item subscales that evaluate cognitive empathy (Perspective‐taking [PT] and Fantasy [FT] subscales), emotional empathy (Empathic Concern [EC] and Personal Distress [PD] subscales). Specifically, PT assesses the ability to understand others’ viewpoints; FT measures the ability to resonate with fictional characters’ feelings; EC evaluates feelings of compassion and concern for others in distress; and PD evaluates feelings of anxiety and discomfort when exposed to others’ negative emotions. One study used only the PD subscale,^43^ one study used only the PT and EC subscales,^56^ and the other three adopted the full IRI to assess overall empathy.

### 3.3 Social cognition differences between people with MCI and dementia

#### 3.3.1 Emotion recognition

##### AD dementia versus MCI

Twenty‐one studies compared facial emotion recognition between people with MCI and AD dementia, with nine reporting better performance in MCI than AD dementia in overall emotion recognition,^51^, ^54^, ^56^, ^57^, ^58^, ^60^, ^64^, ^68^, ^69^, ^76^ six studies finding no significant difference,^52^, ^53^, ^56^, ^61^, ^63^, ^68^ and five studies not conducting between groups statistical analyses.^54^, ^62^, ^74^, ^75^ In one study, the MCI group performed better than the dementia group in the emotion recognition test using whole‐face stimuli, but there were no significant differences in the two groups’ performances in RMET.^78^ For studies that used non‐facial recognition paradigms, one study employing point‐light animation stimuli found that MCI participants performed significantly better than those with AD dementia in recognizing emotions from the point‐light simulated whole‐body figure.^68^ Another study using the emotional prosody test (voice stimuli) also reported significantly better performance in MCI compared to AD dementia,^59^ whereas a different study using the same paradigm found no significant differences between the two groups.^67^

**FIGURE 1:**
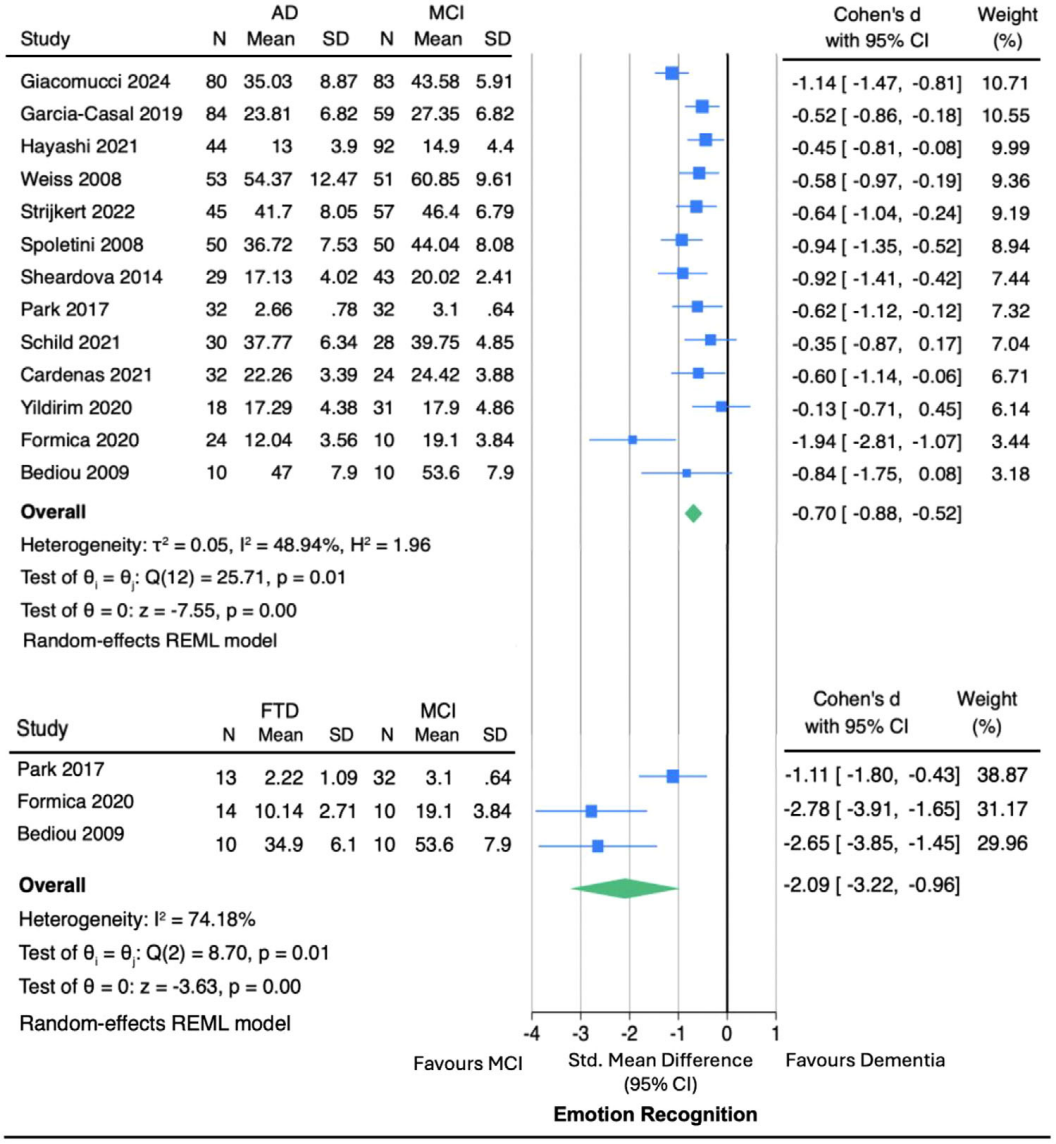
1 Meta‐analysis of emotion recognition in AD dementia versus MCI and FTD versus MCI. The size of the data markers corresponds to the weight of the study. Standardized mean difference was measured by Cohen's *d*, indicating the magnitude of the difference between the two groups. Favors MCI means MCI performed better than dementia. AD, Alzheimer's disease dementia; CI, confidence interval; FTD, frontotemporal dementia; MCI, mild cognitive impairment; N, number; REML, restricted maximum likelihood; SD, standard deviation.

Our meta‐analysis (Figure 1) of 13 studies that adopted the facial emotion recognition paradigm included a total of 531 people with AD dementia and 570 people with MCI. The pooled effect size (Cohen's *d*) was –0.70 (95% Confidence Interval (CI): –0.88 to –0.52) for AD dementia versus MCI. Moderate heterogeneity was observed among the studies (I^2^ = 48.9%). To reduce potential heterogeneity in the analysis, we did not include studies using other modalities, such as voice‐based emotion recognition.

##### AD dementia versus aMCI

The subgroup analysis comparing the emotion recognition between people with AD dementia and aMCI showed similar results (Appendix D). The aMCI group performed better on the emotion recognition tasks than those with AD dementia and no heterogeneity was observed (Cohen's d = –0.61, 95% confidence interval (CI): –0.76 to –0.47; I^2^ = 0.0%).

##### FTD versus MCI

Three studies compared emotion recognition in FTD and MCI using the facial emotion recognition paradigm. Two found that people with MCI performed better than those with FTD,^52^, ^76^ and one study had no inferential statistics on MCI versus FTD comparison.^56^ Our meta‐analysis (Figure 1) of these studies, including 37 FTD participants (8 bvFTD, 5 semantic FTD, 24 subtypes not specified) and 52 MCI participants, found a pooled effect size of –2.09 (95% CI: –3.22 to –0.96), indicating a large effect favoring better emotion recognition performance in the MCI group compared to the FTD group. There was substantial heterogeneity (I^2^ = 74.2%), indicating high variability in the true effect sizes across these studies.

#### 3.3.2 ToM

##### AD deversus MCI

**FIGURE 2:**
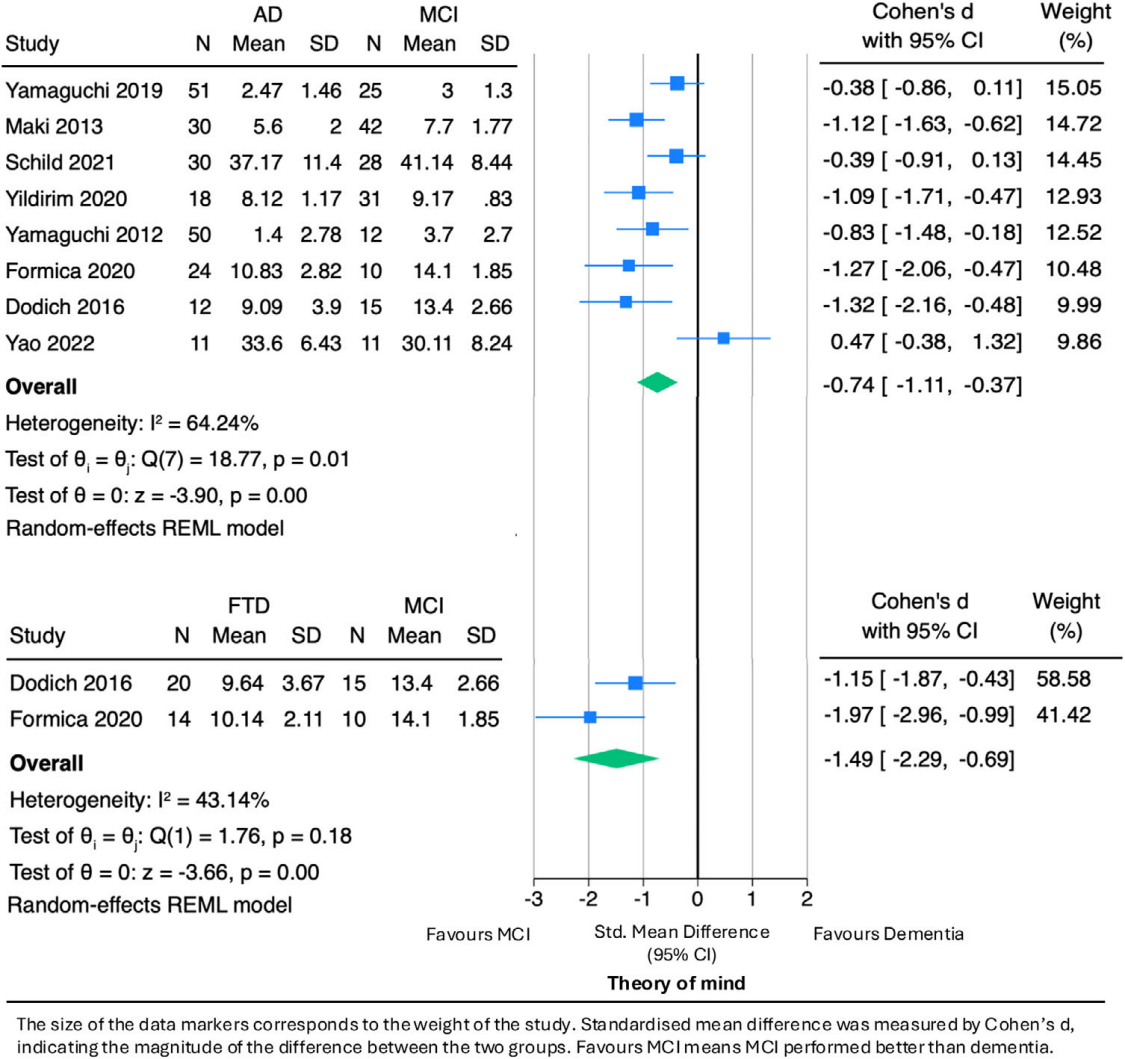
Meta‐analysis of ToM in AD dementia versus MCI and FTD versus MCI. The size of the data markers corresponds to the weight of the study. Standardized mean difference was measured by Cohen's *d*, indicating the magnitude of the difference between the two groups. Favors MCI means MCI performed better than dementia. AD, Alzheimer's disease dementia; CI, confidence interval; FTD, frontotemporal dementia; MCI, mild cognitive impairment; N, number; REML, restricted maxmimum likelihood; SD, standard deviation; ToM, Theory of Mind.

Nine studies compared ToM differences between individuals with AD dementia and MCI. Four of these studies found that individuals with MCI performed significantly better than those with AD dementia in ToM tasks,^53^, ^71^, ^73^, ^76^ three reported no significant differences,^63^, ^66^, ^70^ and two did not make statistical comparisons.^72^, ^74^ Our meta‐analysis (Figure 2) included eight studies, including 226 AD dementia and 174 MCI participants (one study was excluded because of unavailable raw scores). The overall pooled effect size was –0.74 (95% CI: –1.11 to –0.37), indicating a medium to large effect favoring better ToM performance in MCI compared to AD dementia. Substantial heterogeneity was observed among the studies (I^2^ = 64.3%).

##### AD dementia versus aMCI

The pooled effect size (Cohen's *d*) was –0.91 (95% CI: –1.24 to –0.58) for AD dementia versus aMCI, indicating a moderate to large effect favoring higher ToM performance in the aMCI group, with moderate heterogeneity observed (I^2^ = 33.5%) (Appendix D).

##### FTD versus MCI

Two studies compared ToM ability using the SET in 34 people with FTD (including 20 bvFTD and 14 unspecified FTD) and 25 with MCI.^73^, ^76^ Both studies found that MCI participants performed significantly better than FTD participants in ToM tasks. In our meta‐analysis (Figure 2), the pooled effect size was –1.49 (95% CI: –2.29 to –0.69), indicating a large effect. Moderate heterogeneity was observed between the studies (I^2^ = 43.1%).

#### 3.3.3 Empathy

Five studies assessed empathy using the IRI.^51^, ^64^, ^65^, ^73^, ^78^ Three studies found no significant differences in cognitive or emotional empathy between MCI and AD dementia groups.^51^, ^64^, ^73^ However, MCI participants scored higher than FTD participants on the global IRI score and cognitive empathy in the one study.^73^ One reported higher levels of emotional contagion measured by the IRI‐PD subscale (emotional empathy) in dementia compared to MCI.^65^ In addition, one study found better cognitive and emotional empathy ability in people with MCI compared to dementia measured by the IRI‐PT and IRI‐CE subscales, respectively.^78^

**FIGURE 3:**
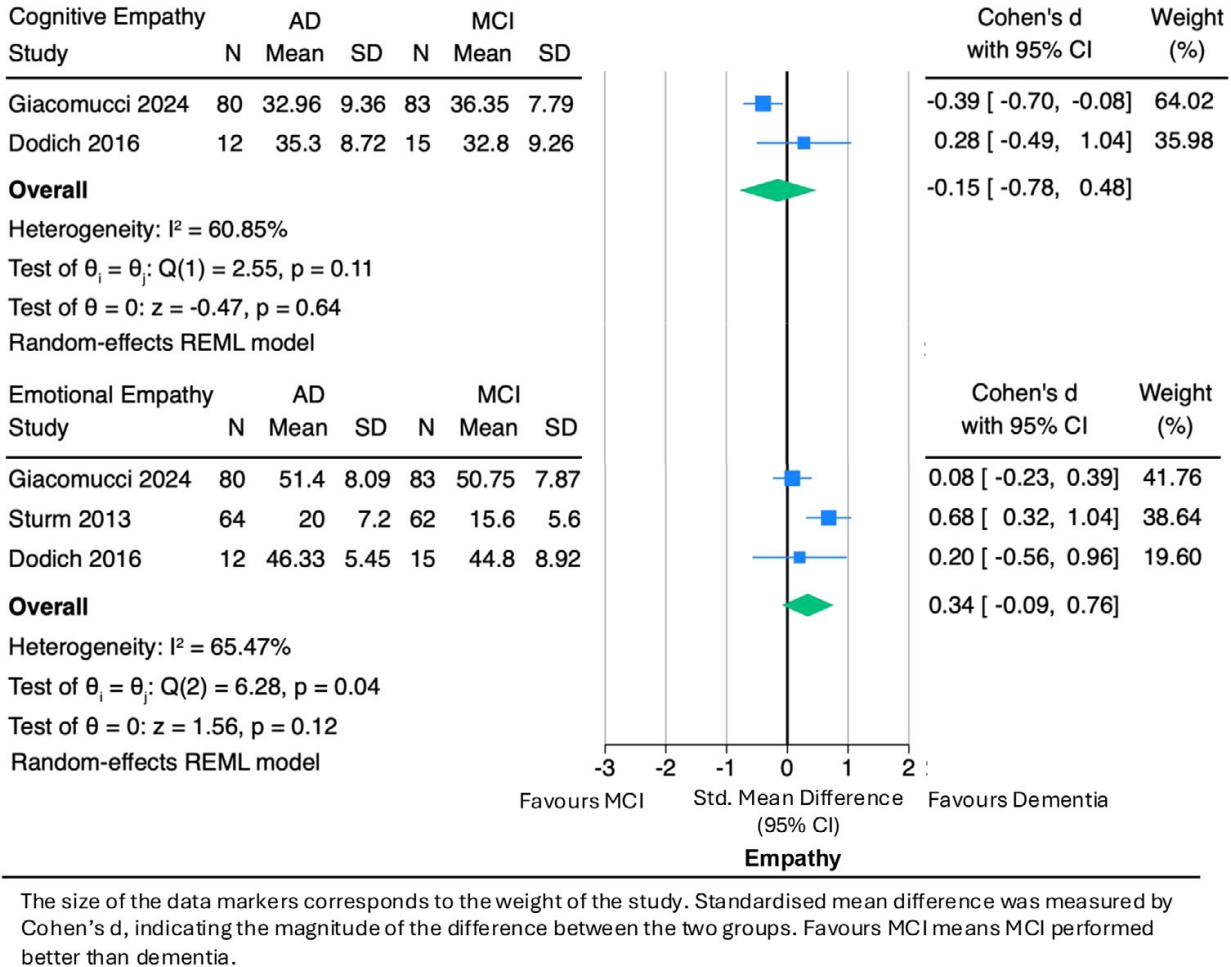
Meta‐analysis of cognitive empathy and emotional empathy in AD versus MCI. The size of the data markers corresponds to the weight of the study. Standardized mean difference was measured by Cohen's *d*, indicating the magnitude of the difference between the two groups. Favors MCI means MCI performed better than dementia. AD, Alzheimer's disease dementia; CI, confidence interval; MCI, mild cognitive impairment; N, number; REML, restricted maximum likelihood; SD, standard deviation.

We conducted two separate meta‐analyses to evaluate differences in cognitive empathy and emotional empathy between the MCI and AD dementia groups (Figure 3). The two studies on cognitive empathy included 92 people with AD dementia and 98 with MCI, and they found a pooled effect size of –0.15 (95% CI: –0.78 to 0.48) and moderate heterogeneity (I^2^ = 60.9%). Our meta‐analysis of emotional empathy between MCI and AD dementia comprised 156 AD dementia and 160 MCI participants. The overall pooled effect size was 0.34 (95% CI: –0.09 to 0.76), and there was moderate heterogeneity (I^2^ = 65.5).

## 4 DISCUSSION

Our systematic review and meta‐analyses of studies examining social cognition in people with MCI and dementia found significant differences in emotion recognition and ToM abilities. People with MCI demonstrated better performance in emotion recognition and ToM compared to those with AD dementia. When comparing MCI to FTD, we found larger effect sizes than in MCI versus AD dementia comparisons, indicating that FTD has a more pronounced effect than AD dementia on social cognition. Empathy differences between MCI and AD dementia were less clear. There was a trend of greater emotional empathy for people with AD dementia than people with MCI, but the pooled difference was not significant. There was also no significant difference in cognitive empathy between the two groups. We were unable to compare empathy between MCI and FTD due to insufficient studies available.

In previous reviews and meta‐analyses of social cognition differences between people with MCI and dementia, researchers have focused only on the AD dementia subtype. One review identified only a small number of studies (emotion recognition:*n* = 4),^32^ whereas another examined only ToM,^34^ and included the RMET as a measure of ToM, which may be more closely related to emotion recognition paradigms.^47^ Our review reports findings that are broadly consistent with but add to previous studies with a comprehensive search strategy, quality rating, and consideration of multiple social cognitive domains and dementia subtypes. However, it is important to note that social cognition is not limited to the three domains of emotion recognition, ToM, and empathy. Social cognition is a dynamic and complex process involving additional cognitive processes that facilitate people in their responses to perceived social information, such as social decision‐making.^12^ Although our search terms looked broadly, the studies identified focused primarily on these three domains and reported specific assessments for these domains. Future research should explore other aspects of social cognition, using tasks that capture its dynamic and context‐dependent nature, to provide a more comprehensive understanding of the social cognition challenges faced by people with dementia.

Our findings of impaired social cognitive abilities in people with dementia, particularly in emotion recognition and ToM, align with our current understanding of neurodegeneration and atrophy in specific regions as dementia progresses. In bvFTD, atrophy affects key regions of social cognition networks, including the insula, anterior cingulate cortex, orbitofrontal cortex, and temporoparietal junction.^13^ In AD dementia, atrophy initially impacts the hippocampus and later extends to the wider temporal, parietal, and frontal cortices.^106^ Several included studies also provided supporting evidence for these neural correlates. For instance, Park et al.^52^ found associations between poorer emotion recognition ability and reduced gray matter volumes in frontal and temporal regions across samples of people with MCI, AD dementia, and FTD, and Sturm et al. ^65^ conducted whole‐brain structural magnetic resonance imaging (MRI) analyses that suggested that smaller volumes in right‐hemisphere temporal lobe structures were associated with heightened negative emotional contagion, which may contribute to emotion dysregulation.

The observed deficits in emotion recognition and ToM abilities in people with dementia will likely result in difficulties in understanding others’ intentions and emotions, and responding appropriately in social interactions, which has the potential to cause distress for both patients and caregivers. As social cognitive abilities decline with progression from MCI to dementia, more prominent social functioning deficits may emerge, and this association was reported in several included studies. For example, Eramudugolla et al.^75^ found that although both MCI and dementia groups reported reduced social network size, only those with dementia reported increased loneliness, which was associated with poorer emotion recognition performance. In Kessel et al.,^63^ people with AD dementia had social functioning impairment that correlated significantly with emotion perception. In addition, Formica et al.^76^ reported a higher burden among caregivers for people with dementia (AD and FTD) compared to MCI caregivers, along with reduced independence in daily activities for the dementia group. Collectively, these findings support the notion that declining social cognitive abilities on testing are associated with worse real‐life social functioning, reduced independence, increased caregiver burden, and greater loneliness, emphasizing the need for early identification of these social cognitive impairments and intervention to preserve quality of life.

Our meta‐analyses on empathy showed no clear evidence of differences in either cognitive or emotional empathy in AD dementia compared to MCI. There was weak evidence of higher emotional empathy in AD dementia compared to MCI, suggesting that empathy abilities may be preserved or even increased in AD dementia. In particular, the study with the largest effect size in our emotional empathy meta‐analysis reported higher emotional empathy (IRI‐PD), reflecting heightened emotional reactivity to negative emotions, which may contribute to emotion dysregulation in AD dementia.^43^ This aligns with recent findings showing that Aβ‐positive dementia participants had higher emotional Interpersonal Reactivity Index‐empathic concern (IRI‐EC) but lower cognitive empathy (IRI‐PT) than Aβ‐negative healthy controls, with greater tau burden in the entorhinal cortex associated with higher EC.^106^ Furthermore, a longitudinal analysis indicated that increases in emotional empathy, measured by IRI empathetic concern, may occur even in the preclinical phase of AD among Aβ‐positive individuals.^107^ However, it should be noted that our meta‐analysis for empathy showed no significant differences, and the small number of included studies limits the strength of these conclusions. In addition, although previous evidence suggests a potential link between increased empathy and positive Aβ markers, our analysis does not directly support this association, especially since the included study with the largest effect size did not report Aβ information. Furthermore, empathy is a dynamic and situation‐dependent process that interacts with personality traits and social factors. The informant‐rated nature of the scales introduces potential measurement bias and may not capture the multifaceted nature of empathy. Overall, the empathy difference between MCI and AD dementia remains unclear, and future studies reporting Aβ markers and using task‐based assessment of empathy are needed.

Our findings indicate that people with dementia exhibit worse performance in emotion recognition and ToM compared to those with MCI. This is consistent with earlier studies showing deficits in these domains among MCI individuals compared to healthy older adults.^35^, ^37^ In addition, there is evidence that social cognitive impairments can emerge long before formal diagnosis in presymptomatic genetic FTDs.^30^, ^31^ These findings support the notion that these social cognition domains decline as dementia progresses and are differentially affected in MCI, AD dementia, and FTD. These results contribute to the current understanding of the social cognitive profile along the dementia continuum. A possible clinical implication is that social cognition deficits at early stages of the disease, such as MCI, may help distinguish MCI cases at higher risk of progression to dementia from stable MCI cases.

However, most social cognition tests have not been examined across healthy control and disease samples to allow us to infer whether an individual's test performance is in the impaired or normal range, thereby impairing current ability to use social cognitive testing in diagnosis. Some studies have normative data that can be used to determine where participants in some studies included in our review lie across population norms. For example, in one included study of emotion recognition, participants with MCI were on average in the 17th to 21st centiles and participants with dementia in the 1st centile compared to a sample of healthy over 60s on the RMET.^76^, ^108^ To enable appropriate use of social cognition testing in clinical settings, future studies should attempt to define normal, borderline, and abnormal test performance.

### 4.1 Limitations

Our study has potential limitations related to the included studies. First, it is unclear to what extent the observed differences are due to social cognition impairments or whether people with dementia performed poorly due to difficulties comprehending and/or remembering task instructions because of general cognitive impairment. Some studies addressed this by including a nonsocial control task to assess general cognitive or visual ability. For example, a study of facial emotion recognition included a task assessing participants’ ability to identify if two faces were the same,^55^ whereas another tested participants’ ability to distinguish the gender of the presented faces.^56^ For ToM, the SET involved a control test assessing participants’ ability to make causal inferences.^73^, ^76^ The Faux Pas Recognition Test also included control questions to assess participants’ general understanding and identification of the situation described in the story.^53^, ^100^ However, most studies did not include such nonsocial control tasks.

Second, there were limitations related to the diagnosis of AD dementia and MCI populations in the included studies. Among participants with AD dementia, Aβ markers were not thoroughly examined (only 279 of 887 AD diagnoses included Aβ information). As a result, the findings may not fully reflect biomarker‐defined AD populations, which are emphasized increasingly in research and clinical trials. For MCI participants in the included studies, most of them had aMCI, which is a subtype linked closely with progression to AD dementia.^85^ As a result, comparisons between FTD and aMCI primarily reflect differences between FTD and prodromal AD dementia, rather than capturing the social cognitive changes along the FTD continuum. There were a small number of naMCI cases in the included studies, but the lack of information on the neuropathology and cognitive domains affected makes it unclear whether these naMCI cases may relate to FTD. Therefore, we were unable to conduct subgroup analyses comparing how these naMCI subtypes differ from AD dementia and FTD in terms of social cognitive impairments. Nonetheless, comparisons between FTD and MCI support the more profound social cognitive impairments in the FTD dementia subtype.

Third, all studies included in our systematic review were cross‐sectional. To address the predictive role of social cognition deficits in dementia progression, longitudinal cohort studies measuring social cognition in MCI and following participants to determine who develops dementia are required, but no such studies were identified in our review.

Finally, some heterogeneity was observed in the analyses, likely stemming from different stages of illness and the variety of assessment methods used. Regarding dementia stages, 12 studies did not specify the stages of AD dementia in their participants, and only three studies reported social cognition performance across different stages of dementia (mild vs moderate AD). Due to the limited data, it was not feasible to conduct subgroup analyses to compare social cognition impairment profiles across dementia stages. As a result, data from different AD dementia stages may have been mixed within the analyses conducted for each social cognition domain, potentially contributing to heterogeneity. For the assessment method, although all studies in the emotion recognition meta‐analysis adopted facial recognition paradigms, they differed in task difficulty and stimuli presentation (e.g., whole face vs partial face in the RMET). For ToM assessment, almost all studies used a different task to assess ToM, each different in their cognitive demands required (e.g., working memory, visual abilities, language comprehension). Moreover, performance on some more complex ToM tasks (e.g., SET) may not exclusively reflect ToM ability, as they also require participants to engage other social cognition abilities, such as emotion recognition, empathy, and social decision‐making. For empathy, we followed the standard categorization of cognitive and affective empathy when the IRI had been used, although the subscales have limitations in measuring these underlying constructs. For cognitive empathy, the IRI‐PT subscale overlaps conceptually with ToM measures, as it primarily assesses the ability to adopt others’ viewpoints. The IRI‐FT subscale has demonstrated poor convergent validity with other cognitive empathy measures.^48^ For affective empathy, the PD subscale may also reflect emotional control or anxiety rather than purely empathy ability. The variability in methodologies and the conceptual overlaps introduces heterogeneity into the meta‐analysis, and the effect sizes and conclusions about social cognition differences across groups should be interpreted with these limitations in mind.

### 4.2 Conclusion and future research

Our systematic review and meta‐analyses demonstrate significant differences in emotion recognition and ToM abilities between people with MCI and those with dementia. Specifically, emotion recognition and ToM abilities show a clear pattern of decline from MCI to AD dementia, with even more pronounced deficits in FTD. Empathy abilities appear to be relatively preserved in people with AD dementia. Further research is needed to clarify how different aspects of empathy are affected in people with distinct dementia syndromes, and to explore whether the observed trend toward increased emotional empathy in AD dementia might be a characteristic feature of the disease.

Our findings strengthen the evidence that social cognition declines as dementia progresses, with potential implications for early detection and intervention. A nuanced assessment of social cognitive abilities in MCI may have clinical value in distinguishing those who will progress to dementia from those who will not, but longitudinal cohort studies of people with MCI are urgently required to confirm the predictive role of the observed social cognition differences. Future studies should assess and compare social cognition across different dementia stages to better understand the impairment profiles in each domain at various stages of the disease (mild, moderate, and severe). There is also a need for more consistent methods of assessing social cognition in dementia, as standardized and widely accepted tools are still lacking and normal range of these tests should be defined. Further studies are needed to compare social cognitive profiles across different MCI subtypes (e.g., aMCI vs non‐aMCI) and their relationship to different dementia outcomes. Clinicians should be aware of the expected social cognitive deficits in people with dementia, assess for these deficits, and advise patients and their families on how to manage these symptoms. In addition, intervention studies are needed to mitigate the impact of dementia on social cognition and to strengthen social relationships.

## CONFLICT OF INTEREST STATEMENT

The authors declare no conflicts of interest. Author disclosures are available in the Supporting Information.

## Supporting information



Supporting Information

Supporting Information

Supporting Information

Supporting Information

Supporting Information
