# Supplementary material for: Social cognition in mild cognitive impairment and dementia: A systematic review and meta‐analysis
Source: Alzheimers Dement. 2025 Mar 27;21(3):e70076. doi: 10.1002/alz.70076 (PMC11947743; doi:10.1002/alz.70076)
Supplement: Supplementary file 1 — Supporting Information [file ALZ-21-e70076-s002.docx]

**Appendix A. Search Terms**

Table A.1. search terms from Ovid MEDLINE (Epub Ahead of Print, In-Process & Other Non-Ovid MEDLINE Epub Ahead of Print, In-Process & Other Non-Indexed Citations, Ovid MEDLINE€ Daily and Ovid MEDLINE 1946 to Present)

| **PIECOS criteria** | **Search Terms** | **Number of papers found** |
| --- | --- | --- |
| **Exposure** | 1 exp Dementia/  2 dementia.mp.  3 Alzheimer* .mp.  4 exp Alzheimer Disease/  5 vascular dementia.mp.  6 exp Frontotemporal Lobar Degeneration/ 7 frontotemporal lobar degeneration.mp.  8 exp Frontotemporal Dementia/  9 frontotemporal dementia.mp.  10 lewy bod*.mp.  11 lewy body dementia.mp.  12 exp Lewy Body Disease/ 13 lewy body disease.mp.  14 exp Dementia, Multi-Infarct/  15 exp Dementia, Vascular/  16 multi infarct dementia.mp.  17 primary progressive aphasia.mp.  18 exp Aphasia, Primary Progressive/  19 presenile dementia.mp.  20 senile dementia.mp. | 210869  164890  209361  122625  8221  6505  3541  5187  10128  12985  1341  4432  5326  1090  7611  919  1833  1207  605  2999 |
|  | 21 - 1 or 2 or 3 or 4 or 5 or 6 or 7 or 8 or 9 or 10 or 11 or 12 or 13 or 14 or 15 or 16 or 17 or 18 or 19 or 20 | 335432 |
| **Comparator** | 22 MCI.mp.  23 Mild Cognitive Impairment.mp.  24 Mild Cognitive*.mp.  25 Mild Neurocognitive*.mp.  26 cognitive impairment.mp.  27 benign senescent forgetfulness.mp.  28 age associated cognitive decline.mp.  29 age associated memory decline.mp.  30 age related cognitive decline.mp.  31 - 22 or 23 or 24 or 25 or 26 or 27 or 28 or 29 or 30 | 24271  25834  27069  346  86866  18  204  235  1763  98472 |
| **Social cognition** | 32 emotion recognition.mp.  33 emotion recogn*.mp.  34 emo* recogn*.mp.  35 emo* perception.mp.  36 emo* perception*.mp. | 5045  5039  5359  1680  1756 |
|  | 37 exp Empathy/  38 empathy.mp.  39 empath*.mp.  40 exp “Theory of Mind”/  41 theory of mind.mp.  42 exp Social cognition/  43 social cognition.mp.  44 social cogn*.mp.  45 social function*.mp.  46 Social Behavior/  47 social behavi*.mp.  48 social activit*.mp.  49 - 32 or 33 or 34 or 35 or 36 or 37 or 38 or 39 or 40 or 41 or 42 or 43 or 44 or 45 or 46 or 47 or 48 | 23255  33754  37675  3765  7428  632  10146  16462  18887  296897 / 58519  75080  7713  383510 / 155072 |
| **AND** | 50 - 21 and 31 | 44614 |
| **AND** | 51 – 49 and 50 | 501 |
| **LIMIT** | Humans | 403 |
| - .mp. = title, abstract, original title, name of substance word, subject heading word, keyword, heading word, protocol supplementary concept word, rare disease supplementary concept word, unique identifier, synonyms - / = Subject heading - exp = explode | | |

Table A.2. Search terms from APA PsychInfo 1806 to January Week 3, 2024.

| **PIECOS criteria** | **Search Terms** | **Number of papers found** |
| --- | --- | --- |
| **Dementia** | 1 dementia.mp.  2 exp Dementia/  3 Alzheimer*.mp.  4 exp Alzheimer’s Disease/  5 vascular dementia.mp.  6 exp Vascular Dementia/  7 frontotemporal lobar degeneration.mp.  8 exp Frontotemporal Lobar Degeneration/  9 frontotemporal dementia.mp.  10 semantic dementia.mp.  11 exp Semantic Dementia/  12 lewy body.mp.  13 lewy bodies.mp.  14 lewy body dementia.mp.  15 dementia with lewy bodies.mp.  16 exp Dementia with Lewy Bodies/ 17 lewy body disease.mp.  18 multi infarct dementia.mp.  19 primary progressive aphasia.mp.  20 exp Aphasia/  21 presenile dementia.mp.  22 exp Presenile Dementia/  23 senile dementia.mp.  24 exp Senile Dementia/ | 89859  93787  78600  56750  4417  2399  1823  3285  5069  3328  3078  3045  4197  658  3346  2462  1897  642  1343  19464  458  295  2164  1116 |
|  | 25 - 1 or 2 or 3 or 4 or 5 or 6 or 7 or 8 or 9 or 10 or 11 or 12 or 13 or 14 or 15 or 16 or 17 or 18 or 19 or 20 or 21 or 22 or 23 or 24 | 148844 |
| **Mild cognitive impairment** | 26 MCI.mp.  27 mild cognitive impairment.mp.  28 exp Mild Cognitive Impairment/  29 mild cognitive*.mp.  30 mild neurocognitive*.mp.  31 cognitive impairment.mp.  32 exp Cognitive Impairment/  33 benign senescent forgetfulness.mp.  34 age associated cognitive decline.mp.  35 age associated memory impairment.mp.  36 age related cognitive decline.mp.  37 age related memory impairment.mp. | 9684  15082  9904  15689  236  64985  45443  28  110  194  1059  99 |
|  | 38 – 26 or 27 or 28 or 29 or 30 or 31 or 32 or 33 or 34 or 35 or 36 or 37 | 66811 |
| **Social cognition** | 39 emotion* recognition.mp.  40 exp Emotion Recognition/  41 emotion* perception.mp.  42 empathy.mp.  43 exp Empathy/  44 empath*.mp.  45 theory of mind.mp.  46 exp “Theory of Mind”/  47 social cognition.mp.  48 exp Social cognition/  49 social* cogn*.mp.  50 social* function*.mp.  51 social* behav*.mp.  52 Social Behavior/  53 social activities.mp.  54 social activity.mp. | 7369  4553  2098  37572  17293  44638  11984  7149  23423  19728  33718  26571  99498  20242  4102  2395 |
|  | 55 – 39 or 40 or 41 or 42 or 43 or 44 or 45 or 46 or 47 or 48 or 49 or 50 or 51 or 52 or 53 or 54 | 213455 |
| **AND** | 56 – 25 and 38  57 – 55 and 56 | 30411  528 |
| **LIMIT** | 58 Limit 57 to Human | 494 |
| - .mp. = title, abstract, original title, name of substance word, subject heading word, keyword, heading word, protocol supplementary concept word, rare disease supplementary concept word, unique identifier, synonyms - / = Subject heading - exp = explode | | |

Table A.3. Search terms for Embase 1974 to 22 January, 2024.

| **PIECOS criteria** | **Search Terms** | **Number of papers found** |
| --- | --- | --- |
| **Dementia** | 1 dementia.mp.  2 exp Dementia/  3 Alzheimer*.mp.  4 exp Alzheimer’s Disease/  5 vascular dementia.mp.  6 exp Vascular Dementia/  7 frontotemporal lobar degeneration.mp.  8 exp Frontotemporal Lobar Degeneration/  9 frontotemporal dementia.mp.  10 semantic dementia.mp.  11 exp Semantic Dementia/  12 lewy body.mp.  13 lewy bodies.mp.  14 lewy body dementia.mp.  15 dementia with lewy bodies.mp.  16 exp Dementia with Lewy Bodies/ 17 lewy body disease.mp.  18 multi infarct dementia.mp.  19 primary progressive aphasia.mp.  20 exp Aphasia/  21 presenile dementia.mp.  22 exp Presenile Dementia/  23 senile dementia.mp.  24 exp Senile Dementia/ | 89859  93787  78600  56750  4417  2399  1823  3285  5069  3328  3078  3045  4197  658  3346  2462  1897  642  1343  19464  458  295  2164  1116 |
|  | 25 - 1 or 2 or 3 or 4 or 5 or 6 or 7 or 8 or 9 or 10 or 11 or 12 or 13 or 14 or 15 or 16 or 17 or 18 or 19 or 20 or 21 or 22 or 23 or 24 | 148844 |
| **Mild cognitive impairment** | 26 MCI.mp.  27 mild cognitive impairment.mp.  28 exp Mild Cognitive Impairment/  29 mild cognitive*.mp.  30 mild neurocognitive*.mp.  31 cognitive impairment.mp.  32 exp Cognitive Impairment/  33 benign senescent forgetfulness.mp.  34 age associated cognitive decline.mp.  35 age associated memory impairment.mp.  36 age related cognitive decline.mp.  37 age related memory impairment.mp. | 9684  15082  9904  15689  236  64985  45443  28  110  194  1059  99 |
|  | 38 – 26 or 27 or 28 or 29 or 30 or 31 or 32 or 33 or 34 or 35 or 36 or 37 | 66811 |
| **Social cognition** | 39 emotion* recognition.mp.  40 exp Emotion Recognition/  41 emotion* perception.mp.  42 empathy.mp.  43 exp Empathy/  44 empath*.mp.  45 theory of mind.mp.  46 exp “Theory of Mind”/  47 social cognition.mp.  48 exp Social cognition/  49 social* cogn*.mp.  50 social* function*.mp.  51 social* behav*.mp.  52 Social Behavior/  53 social activities.mp.  54 social activity.mp. | 7369  4553  2098  37572  17293  44638  11984  7149  23423  19728  33718  26571  99498  20242  4102  2395 |
|  | 55 – 39 or 40 or 41 or 42 or 43 or 44 or 45 or 46 or 47 or 48 or 49 or 50 or 51 or 52 or 53 or 54 | 213455 |
| **AND** | 56 – 25 and 38  57 – 55 and 56 | 30411  528 |
| **LIMIT** | 58 Limit 57 to Human | 494 |
| - .mp. = title, abstract, original title, name of substance word, subject heading word, keyword, heading word, protocol supplementary concept word, rare disease supplementary concept word, unique identifier, synonyms - / = Subject heading - exp = explode | | |

Table A.4. Search terms for CINAHL

| **PIECOS criteria** | **Search Terms** | **Number of papers found** |
| --- | --- | --- |
| **Dementia** | S1 (MH “Dementia+”)  S2 dementia  S3 alzheimer’s disease  S4 alzheimer*  S5 (MH “Alzheimer’s Disease”)  S6 vascular dementia  S7 (MH “Dementia, Vascular+”)  S8 frontotemporal lobar degeneration S9 (MH "Frontotemporal Lobar Degeneration+")  S10 frontotemporal dementia  S11 (MH "Frontotemporal Dementia+")  S12 lewy bod*  S13 lewy body dementia  S14 (MH “Lewy Body Disease”)  S15 lewy body disease  S16 dementia, multi-infarct  S17 (MH "Dementia, Multi-Infarct")  S18 primary progressive aphasia  S19 presenile dementia  S20 senile dementia | 85123  79338  51000  53093  38026  3312  1954  1404  1187  2573  1047  3064  2332  1435  2160  1229  308  765  177  31016 |
|  | S21 - S1 OR S2 OR S3 OR S4 OR S5 OR S6 OR S7 OR S8 OR S9 OR S10 OR S11 OR S12 OR S13 OR S14 OR S15 OR S16 OR S17 OR S18 OR S19 OR S20 | 114983 |
| **Mild cognitive impairment** | S22 MCI.mp.  S23 mild cognitive impairment.mp.  S24 mild cognitive*  S25 mild Neurocognitive*  S26 cognitive impairment  S27 benign senescent forgetfulness  S28 age-associated cognitive decline  S29 age associated memory decline  S30 - S22 OR S23 OR S24 OR S25 OR S26 OR S27 OR S28 OR S29 | 7352  10923  11923  208  49381  3  64  29  51425 |
| **Social cognition** | S31 emotion recognition  S32 emotion recogn*  S33 emo* recogn*  S34 emo* perception  S35 emo* perception*.mp. | 1598  1945  2803  2387  2391 |
|  | S36 (MH “Empathy”)  S37 empathy  S38 empath*  S39 theory of mind  S40 (MH “Theory of Mind”)  S41 social cognition  S42 (MH “Social cognition”)  S43 social cogn*  S44 social function*  S45 (MH “Social Behavior+”)  S46 social behavi*  S47 social activit*  S48 - S31 OR S32 OR S33 OR S34 OR S35 OR S36 OR S37 OR S38 OR S39 OR S40 OR S41 OR S42 OR S43 OR S44 OR S45 OR S46 OR S47 | 13563  17790  20050  2508  1218  3292  423  14384  16618  51960 / 25998  44530  11709  125008 / 100664 |
| **AND** | S49 - S21 and S30 | 18783 |
| **AND** | S50 – S48 and S49 | 602 |
| **LIMIT** |  |  |
| - .mp. = title, abstract, original title, name of substance word, subject heading word, keyword, heading word, protocol supplementary concept word, rare disease supplementary concept word, unique identifier, synonyms - / = Subject heading - exp = explode | | |
