# Supplementary figures and images for: Social cognition in mild cognitive impairment and dementia: A systematic review and meta‐analysis
Source: Alzheimers Dement. 2025 Mar 27;21(3):e70076. doi: 10.1002/alz.70076 (PMC11947743; doi:10.1002/alz.70076)

**Appendix B. Prisma Diagram**

**
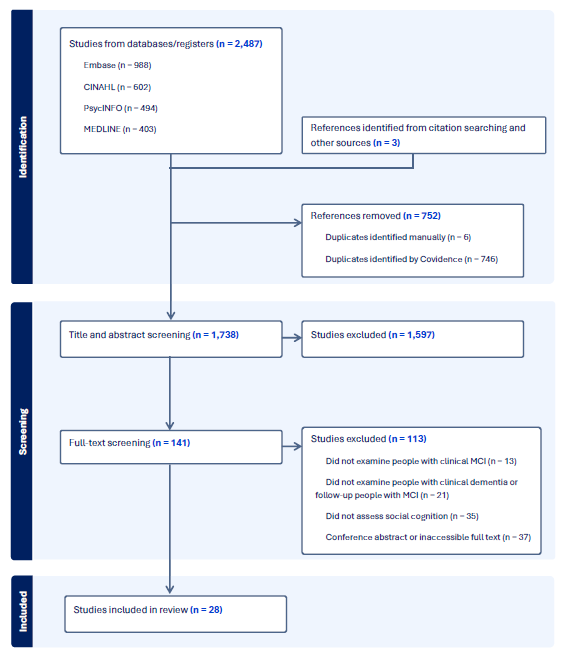
**

Figure B.1 Prisma Diagram

Supplement: Supplementary file 2 — Supporting Information [file ALZ-21-e70076-s005.docx]
