# Supplementary material for: Social cognition in mild cognitive impairment and dementia: A systematic review and meta‐analysis
Source: Alzheimers Dement. 2025 Mar 27;21(3):e70076. doi: 10.1002/alz.70076 (PMC11947743; doi:10.1002/alz.70076)
Supplement: Supplementary file 3 — Supporting Information [file ALZ-21-e70076-s004.docx]

**Appendix C. Data transformations, and reasons for exclusion for meta-analyses**

# Data transformation

Data transformation was conducted in two situations: 1) Some studies reported scores for subgroups of a certain diagnosis but not an overall score (e.g., mild AD and moderate AD; single-domain MCI and multi-domain MCI [whether a single cognitive domain or multiple cognitive domains were affected]). In these cases, the average score and standard deviation were calculated and used in the analysis. 2) Some studies reported social cognition scores for subscales/sub-tasks for the Dementia and MCI groups, but not the overall score of the scales/tasks. Therefore, the combined scores and standard deviations for the overall scale/tasks were calculated.

The specific transformations included:

- Sheardova (2014): Averaged the scores and standard deviations for sd-MCI and md-MCI.
- Weiss (2008): Averaged the scores and standard deviations for mild AD + moderate AD, sd-MCI, and md-MCI.
- Yamaguchi (2012): Averaged the scores and standard deviations for mild AD and moderate AD.
- Yamaguchi (2019): Averaged the scores and standard deviations for mild AD and moderate AD.
- Maki (2013): Combined MSST sub-scale scores to obtain an overall score and standard deviation.
- Cardenas (2021): Added/combined the scores and standard deviations of similar tasks (excluding the prosody task); all participants were moderate AD.
- Giacomucci (2024): The subscales for emotional empathy (EC, PD) and cognitive empathy (FT, PT) were added/combined.
- Schild (2021): Combined the two tasks, RMET and KDEF

# Studies excluded in the meta-analyses and reasons for exclusion

- Ferrer-Cairols et al., 2023: Only the median and range were reported, and mean scores for the social cognition assessment were not available.
- Kessels et al., 2021: Mean scores for social cognition assessment were not available.
- Henry et al., 2012: the mean scores for the biological motion task was not clear (only a graph was provided), and the participants might overlap with those in Henry et al., 2009.
- Giacomucci et al., 2022: The participants might overlap with those in Giacomucci et al., 2024.
- Eramudugolla et al., 2022: The study included participants with diagnoses other than AD and FTD.
- Henry et al., 2009, 2012: Dementia subtype was not specified.
- Hayashi et al., 2021: Dementia subtype was not specified.
- Chander et al., 2024: Dementia subtype was not specified.
