# Supplementary material for: Social cognition in mild cognitive impairment and dementia: A systematic review and meta‐analysis
Source: Alzheimers Dement. 2025 Mar 27;21(3):e70076. doi: 10.1002/alz.70076 (PMC11947743; doi:10.1002/alz.70076)
Supplement: Supplementary file 4 — Supporting Information [file ALZ-21-e70076-s001.docx]

**Appendix D. Forest plots for comparison of social cognition in people with Alzheimer’s disease dementia (AD) to those with amnestic mild cognitive impairment (aMCI)**


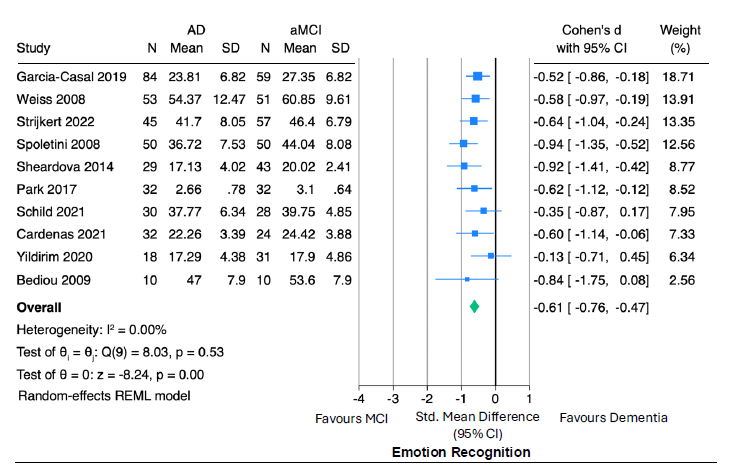


Figure D.1. Meta-Analysis of Emotion Recognition in AD vs aMCI

The size of the data markers corresponds to the weight of the study. Standardised mean difference was measured by Cohen’s d, indicating the magnitude of the difference between the two groups. Favours MCI means MCI performed better than dementia.


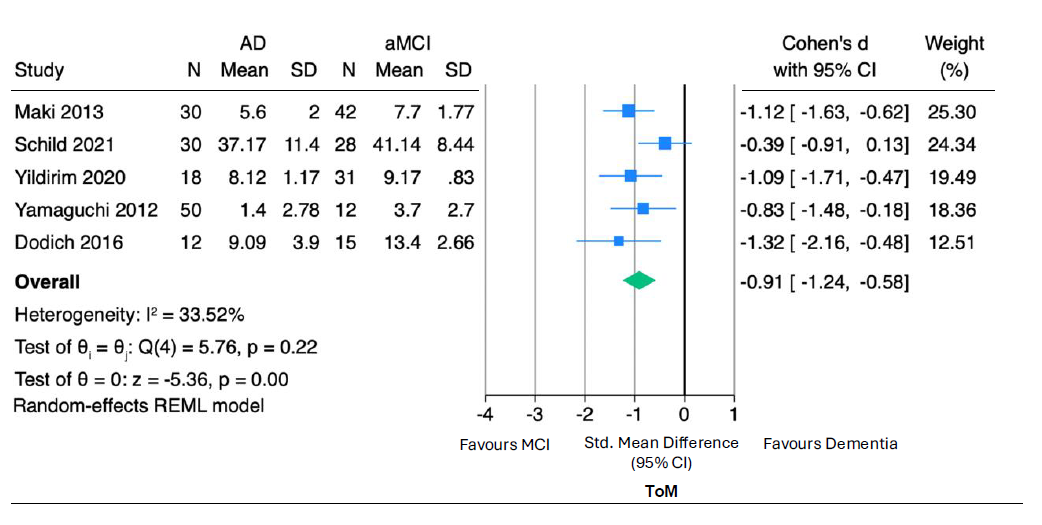


Figure D.2. Meta-Analysis of ToM in AD vs aMCI

The size of the data markers corresponds to the weight of the study. Standardised mean difference was measured by Cohen’s d, indicating the magnitude of the difference between the two groups. Favours MCI means MCI performed better than dementia.
